# Status of nutrients important in brain function in phenylketonuria: a systematic review and meta-analysis

**DOI:** 10.1186/s13023-018-0839-x

**Published:** 2018-06-26

**Authors:** Gina A. Montoya Parra, Rani H. Singh, Aysun Cetinyurek-Yavuz, Mirjam Kuhn, Anita MacDonald

**Affiliations:** 1 Danone Nutricia Research, Nutricia Advanced Medical Nutrition, Utrecht, The Netherlands; 2 Metabolic Genetics and Nutrition Program, Emory University, Atlanta, GA USA; 3 Department of Metabolic Diseases, Birmingham Children’s Hospital, Birmingham, UK

**Keywords:** Phenylketonuria (PKU), Brain, Plasma, Nutrient, Nutritional requirement, Metabolism, Fatty acids, Docosahexaenoic acid (DHA), Eicosapentanoic acid (EPA), Cholesterol, choline, Vitamins, Micronutrients, phospholipids, compliance

## Abstract

**Background:**

Despite early and ongoing dietary management with a phe-restricted diet, suboptimal neuropsychological function has been observed in PKU. The restrictive nature of the PKU diet may expose patients to sub-optimal nutritional intake and deficiencies which may impact normal brain function. A systematic review of the published literature was carried out, where possible with meta-analysis, to compare the status of nutrients (Nutrients: DHA, EPA phospholipids, selenium, vitamins B_6_, B_12_, E, C, A, D, folic acid, choline, uridine, calcium, magnesium, zinc, iron, iodine and cholesterol) known to be important for brain development and functioning between individuals with PKU and healthy controls.

**Results:**

Of 1534 publications identified, 65 studies met the entry criteria. Significantly lower levels of DHA, EPA and cholesterol were found for PKU patients compared to healthy controls. No significant differences in zinc, vitamins B_12_, E and D, calcium, iron and magnesium were found between PKU patients and controls. Because of considerable heterogeneity, the meta-analyses findings for folate and selenium were not reported. Due to an insufficient number of publications (< 4) no meta-analysis was undertaken for vitamins A, C and B_6_, choline, uridine, iodine and phospholipids.

**Conclusions:**

The current data show that PKU patients have lower availability of DHA, EPA and cholesterol. Compliance with the phe-restricted diet including the micronutrient fortified protein substitute (PS) is essential to ensure adequate micronutrient status. Given the complexity of the diet, patients’ micronutrient and fatty acid status should be continuously monitored, with a particular focus on patients who are non-compliant or poorly compliant with their PS. Given their key role in brain function, assessment of the status of nutrients where limited data was found (e.g. choline, iodine) should be undertaken. Standardised reporting of studies in PKU would strengthen the output of meta-analysis and so better inform best practice for this rare condition.

**Electronic supplementary material:**

The online version of this article (10.1186/s13023-018-0839-x) contains supplementary material, which is available to authorized users.

## Background

Phenylketonuria (OMIM 261600) is a rare genetic disorder characterized by impaired conversion of phenylalanine (phe) to tyrosine (tyr) due to deficient activity of the hepatic enzyme L-phenylalanine-4- hydroxylase [1]. If untreated, the resulting high blood phe concentrations cross the blood brain barrier causing detrimental effects on brain development and function [2]. For the majority of patients with PKU, treatment is a phe-restricted diet that aims to prevent excessive accumulation of phe while also meeting requirements for growth and development [3]. Because of the severe restriction of many protein-containing foods, the diet requires supplementation with a phe-free protein substitute (PS) and a wide range of other nutrients including omega-3 fatty acids, vitamins, minerals and trace elements. However, nutrient intakes [4–6] and status [7–9] may be affected by factors such as poor dietary compliance, bioavailability of different nutrient sources, different management approaches in metabolic centres [2] and historical differences in the composition of the PS e.g. changes in selenium fortification practices [2, 4, 10–12]. Despite early and continuous low phe dietary management, PKU patients have been shown to have suboptimal neuropsychological function compared to healthy controls, in particular lower IQ, slower information processing [13, 14] and suboptimal executive functioning [15–17].

Tyr deficiency has been suggested as one reason for the decreased neuropsychological performance and cognitive dysfunction in PKU [18]. It has been suggested that tyr supplementation could have an important role in the treatment of PKU [19], however blood tyr concentrations do not correlate with cognitive outcomes in PKU, tyr supplementation alone does not prevent severe mental retardation [20] and 24 h tyr plasma levels show high variability. Reduced serotonin synthesis may be the result of reduced tryptophan (trp) brain concentrations caused by reduced blood-brain barrier transport of trp at elevated plasma phe concentrations [21, 22]. Different hypotheses focusing on the pathogenesis of PKU and disturbed amino acids transport from blood to brain on cerebral neurotransmitter and protein synthesis have been proposed, a detailed examination of this evidence is beyond the scope of this review.

Under normal physiological conditions, many other nutrients have an important role in brain development and function affecting multiple processes regulating neurotransmitter pathways, synaptic transmission, membrane fluidity and signal-transduction pathways [23]. Table 1 provides a summary of the key role of nutrients involved in brain development and functioning such as structural components of membranes, antioxidants, neurotransmitter precursors and co-factors [23–29]. For many of these nutrients, food sources are restricted in PKU diets (Table 1) and reduced levels of biomarkers for some of these nutrients have been observed in PKU [5, 30–36]. Poor status of some antioxidant nutrients have been associated with impaired cerebral function [30] and neuropsychological disturbances in PKU [37]. Improvements in markers of cognition (visual function and fine motor skills) have been found following supplementation with docosahexaenoic acid (DHA) in children with PKU [31, 38]. In addition to raised phe, sub-optimal levels of some of these nutrients could have an impact on cognition in PKU patients. Given this background, this is the first systematic review to investigate the status of multiple nutrients involved in brain function. Although the review will not investigate the functional outcomes of any altered levels of these nutrients found, there is a rationale that in PKU optimal levels of these nutrients, are key to achieve an optimal cognitive potential. Specifically, we have systematically reviewed, and performed meta-analysis where possible, the nutrient status of DHA, eicosapentanoic acid (EPA), phospholipids, selenium, vitamins B_6_, B_12_, E, C, A, D, folic acid, choline, uridine, calcium, magnesium, zinc, iron, iodine and cholesterol that are important for optimal brain function. This will indicate whether differences in nutrient status exist between individuals with PKU and healthy, non-PKU controls.

**Table 1:** Role of selected nutrients in brain function and their food sources [23, 24]

Nutrient	Role in brain function	Main food sources
DHA, EPA and phospholipids (PL)	DHA is abundant in the brain. DHA and EPA are components of different PL in synaptic cell membranes. Involved in membrane fluidity and function [48, 103].	DHA and EPA: oily fish. PL: soya, rapeseed, sunflower, eggs, milk.
Choline	Component of PL. Precursor of neurotransmitter acetylcholine. Modulates neuronal membrane formation.	Meat, dairy products, grains, eggs and fish.
Uridine	Constituent of nucleotides, nucleic acids, precursor of brain phosphatidylcholine in membranes [104, 105].	Ribonucleic acid form in foods not bioavailable. As uridine monophosphate in human milk. De-novo synthesis to meet requirements.
Cholesterol	Essential component of neuronal membranes [25], involved in signalling, synaptic plasticity, learning and memory [106]. Also converted to bioactive oxysterols and vitamin D [107]	Eggs and fat containing foods.
Vitamin D	Neuro-steroid, modulates neurotransmission. Helps maintain calcium balance and signalling. Contributes to synaptic plasticity [82, 83].	Limited dietary sources; mainly oily fish, egg, fortified foods.
Vitamins A, C and E, selenium, zinc	Critical role as antioxidants in the brain. Zinc also has a role as a neurosecretory product in the synaptic vesicles of specific neurons [108].	Vitamin A: offal, dairy products, eggs, carrots and dark green leafy vegetables. Vitamin C: fruits and vegetables Vitamin E: nuts, seeds, oily fish, egg yolk and whole grain cereals. Selenium: meat, fish, legumes, grains (variable content in soil). Zinc: meat, legumes, eggs, fish, grains.
Calcium	Important intra-cellular brain messenger [109] required for synaptic plasticity and secretion of neurotransmitters.	Dairy products.
Magnesium	Co-factor in multiple enzyme reactions and regulates N-methyl-D-aspartate receptors. Role in release of neuropeptides in the brain [110].	Nuts, whole grains, fish, seafood, several vegetables.
Iron	Important in oxygen transport for optimal cognitive function.	Meat, fish, cereals, legumes, nuts, egg yolks, some vegetables, potatoes and fortified foods.
Vitamins B_6,_ B_12_ and folate	Vitamin B_6_: neurotransmitter synthesis. Vitamin B_12_ and folate: important for oxygen transport for optimal cognitive function. Vitamin B_12_ is involved in myelin synthesis.	Vitamin B_6_: grains, legumes, nuts, seeds, potatoes, meat and fish. Vitamin B_12_: meat (especially offal), fish, dairy products, eggs. Folate: dark green leafy vegetables, legumes, fruits and fortified cereals.
Iodine	Constituent of thyroid hormones. Important in foetal brain development.	Fish, dairy products, eggs and iodized salt.

## Methods

### Search strategy and selection criteria

Literature published from 1990 to July 2016 was systematically searched in the Cochrane Central Register of Controlled Trials, Medline and Embase electronic databases in accordance with the PRISMA guidelines. The nutrients identified of interest were: DHA, EPA phospholipids, selenium, vitamins B_6_, B_12_, E, C, A, D folic acid, choline, uridine, calcium, magnesium, zinc, iron, iodine and cholesterol. To avoid the risk of bias, general search terms were chosen (such as nutrition and diet) to ensure that papers reporting nutrient levels as a secondary outcome would also be included. Further details of the search strategy are presented in the Additional file 1.

**Fig. 1:**
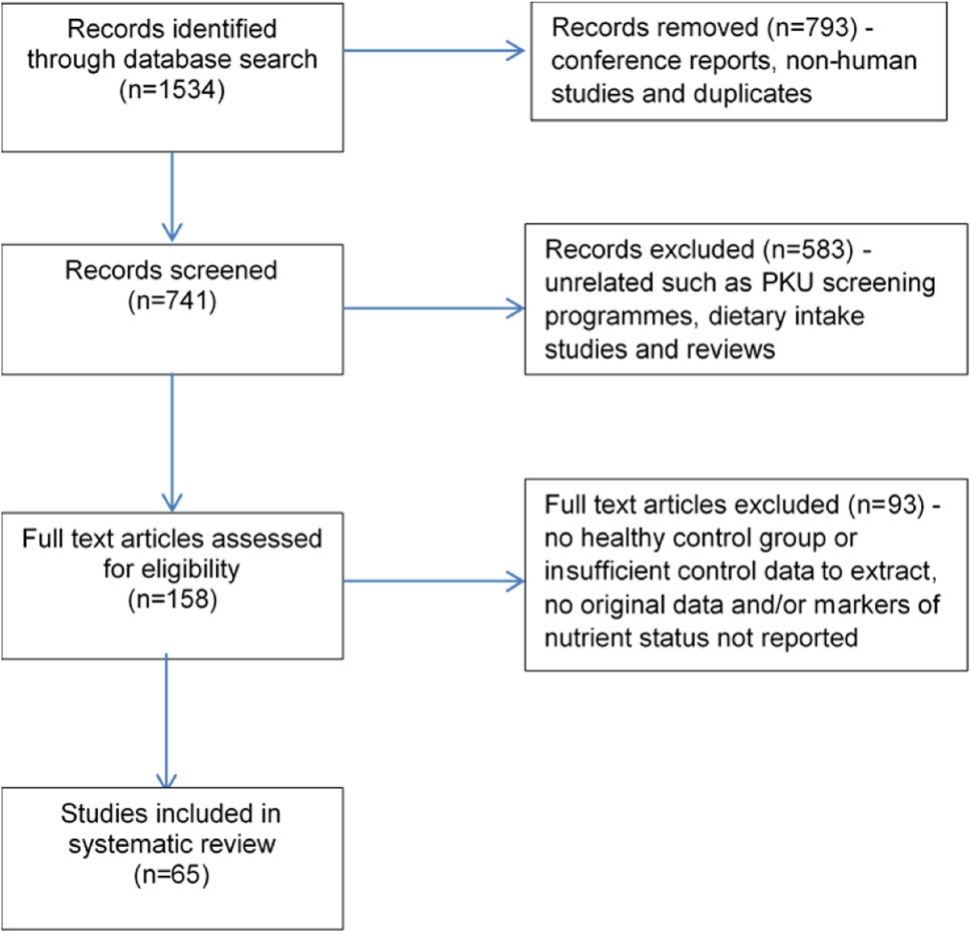
Flowchart of publication search and study selection for the systematic review. Breakdown of the retrieved publications leading to the selection of the 65 publications suitable for systematic review/meta-analysis

The search strategy identified 1534 publications (Fig. 1). Following the removal of conference reports, animal studies and obvious duplicates, the titles and abstracts of the remaining 741 were screened for relevance by a reviewer. Studies that may have contained results of biomarkers of a nutrient of interest, even if not explicitly mentioned in the title or abstract, were retained. One hundred and fifty-eight publications were identified as potentially relevant. Potentially eligible articles that were in a language other than English were translated. A 10% sample of the excluded articles was reviewed by a second reviewer to ensure none had been excluded that may have been relevant to the review.

The full-text publications considered possibly relevant were reviewed against the following inclusion criteria: contained a treated PKU population and healthy controls, observational or intervention study published in a peer-reviewed journal, included extractable data for biomarkers of the nutrients of interest, identified numbers of participants and their characteristics. The exclusion criteria were: untreated patients with PKU, studies in maternal PKU, in-vitro or animal studies and, if reported, studies in which the healthy control group took a vitamin and/or mineral supplement that contained any of the nutrients under review. All of the publications considered potentially relevant were reviewed by a second reviewer to determine if the same conclusion was reached about their suitability for inclusion or exclusion and any disagreements were resolved through discussion. Where the retrieved documents revealed duplication of the same patient population, the publication with the most complete set of data was included.

### Data extraction

Data was extracted from the 65 publications that met the eligibility criteria (Fig. 1). Details of the participant characteristics, plasma phe control and nutrient supplementation, if provided, were extracted and relevant nutrient biomarkers of interest for PKU subjects and healthy controls were extracted from each study into a standard form by one of the reviewers and checked by a second reviewer. Biomarkers for the nutrients under review for all PKU subjects within a study were extracted, irrespective of phe control. Where the PKU population was sub-grouped (e.g. by phe control or baseline intervention group), the data were combined. The only subgroup excluded, where it was possible, was mild PKU (hyperphenylalaninaemia [HPA]), due to their less restrictive diet. Where more than one biomarker of interest for a nutrient was reported, a hierarchical approach was taken i.e. levels in plasma/serum were extracted over levels in erythrocytes, and levels in erythrocytes were extracted over other indicators of nutrient status. The most widely accepted indicator of status was extracted e.g. serum ferritin rather concentrations of iron in serum. For intervention studies, only the baseline data was utilised.

### Statistical analysis

Reported comparisons of nutrient levels in PKU patients and controls were integrated and summarized into a final result per nutrient, using meta-analysis methods [39]. The PRISMA (Preferred Reporting Items for Systematic Reviews and Meta-Analyses) guidelines for the reporting of systematic reviews and meta-analyses were followed in ensuring that an evidence-based minimum set of items were extracted [40]. For each nutrient, the information extracted from the articles included: levels expressed in mean and standard deviation of plasma/serum/other matrices in PKU patients and in controls, number of subjects and the average age per group. Where means and SDs were not reported, these values were estimated from other statistical measures. To allow comparison across studies, the standardized mean difference (SMD) was used to standardize the results of the studies to the same scale, which expresses the size of the effect in each study relative to the variability observed in that study.

These data were analysed using the random-effect meta-analysis model [39] fitted by restricted maximum likelihood estimation (REML) in SAS 9.4 (SAS Institute, Cary NC) using proc. mixed procedure. For this analysis, a minimum of four studies is generally recognized to be required [41, 42]. For parameters with a small number of studies where heterogeneity is not too high, fixed effect meta-analysis has been implemented using proc. mixed procedure in SAS 9.4 (SAS Institute, Cary NC). Due to small number of studies for some nutrients, it is preferred to calculate the 95% confidence intervals using a t-distribution (with a degrees of freedom = number of studies-1) instead of using a normal distribution. Estimate of the between-study variance in a random-effects meta-analysis is presented by Tau^2^ where *p* < 0.05 refers to a considerable amount of heterogeneity, and I^2^ statistic (introduced by Higgins and Thompson [43]) where and I^2^ > 95% indicates considerable amount of heterogeneity.

## Results

Sixty-five publications were identified that provided outcomes for one or more of the nutrients reviewed, and the characteristics of these studies and the PKU populations included are summarized in Table 2.

**Table 2:** Characteristics of the eligible studies

	n
Study design	
Observational studies	58
Intervention studies	7
Countries of origin	
European	48^a^
USA	9
Rest of the world	8
Age groups of subjects	
Children (< 14 yrs) only	24
Children and adolescents (< 19 yrs) only	15
Adults only	7
Mixed age group (infants/children, adolescent and adults)	19
Phentoytpe	
Classical PKU only	26
Classical and moderate PKU	5
Insufficient details to determine sub-classifications of PKU	34
Information on dietary control	
Information on dietary control provided^c^	54
Information on dietary control **not** provided	11
Information on phe-free PS	
Information on PS provided^d^	36
Information on PS **not** provided	29

Notes: ^a^3 Austria, 1 Czech Republic, 2 France, 9 Germany, 7 Greece, 3 Italy, 2 The Netherlands, 2 Poland, 1 Portugal, 1 Russia, 11 Spain, 1 Switzerland, 3 Turkey and 2 UK.

^b^3 Japan, 2 Brazil, 1 Australia, 1 China and 1 Lebanon.

^c^Either details of baseline Phe control at entry provided or the study entry criteria required that patients had to be well-controlled.

^d^Either information detailing the specific PS used by patients or whether a supplementary source of the nutrients that were the subject of this review were taken by PKU subjects.

Data in bold is highly significant

Table 3 summarises the studies included in the systematic review and the differences in the outcomes in nutrient status markers between PKU patients and healthy controls in the individual studies. Where ≥4 studies were retrieved for a nutrient, meta-analyses of the data were performed and the overall effect estimate is presented (SMD with confidence intervals [CI]), illustrated in forest plots), although it should be noted that there was significant heterogeneity within the datasets for all nutrients. When the heterogeneity is high (I^2^ > 95%) and there is no statistically significant difference between the groups the overall conclusion is estimated and presented in the forest plots. Otherwise when heterogeneity is high for a given nutrient, the overall effect is not reported as it would not correctly represent the between group difference. Where < 4 studies were retrieved, the forest plots have been included to provide a graphical display of the data from individual studies which shows to which extent study results overlap without presenting the overall estimate.

**Table 3:** Systematic review results and key outcomes on differences in nutrient status between PKU patients and healthy controls

Nutrient	Number of publications	Number of subjects from retrieved publications		Studies reporting significantly lower levels in PKU compared to controls	Studies reporting significantly higher levels in PKU compared to controls	Studies reporting no significant difference between PKU and controls
		PKU	Controls			
DHA^a, b^	16	479	586	9 studies [5, 32, 111–117]	–	6 studies [52–54, 118–120]
EPA^c^	14	397	513	5 studies [5, 32, 112, 114, 117]	–	8 studies [52–54, 111, 113, 115, 119, 120]
Choline	2	24	41	1 study [33]	–	1 study [98]
Cholesterol^d^	18	727	837	12 studies [55, 61, 107, 111, 121–128]	–	6 studies [53, 86, 112, 119, 129, 130]
Uridine	0	–	–	–	–	–
PL	2	26	22	1 study [34]	–	1 study [112]
Vitamin A	3	191	190	–	–	3 studies [44, 131, 132]
Vitamin E^d^	7	339	346	1 study [131]	1 study [126]	5 studies [30, 35, 37, 44, 132]
Vitamin C^d^	3	138	168	–	1 study [132]	2 studies [44, 126]
Selenium	16	666	618	14 studies [6, 30, 35, 44, 67, 69–73, 132–135]	–	2 studies [37, 45]
Vitamin B_6_^d^	2	89	56	1 study [136]	1 study [137]	–
Vitamin B_12_^d^	6	307	1883	1 study [136]	3 studies [85, 119, 138]	2 studies [88, 89]
Folic acid^d^	7	306	1817	1 study [136]	5 studies [85, 87–89, 138]	1 study [86]
Calcium^d^	11	373	610	2 studies [76, 133]	1 study [139]	8 studies [77, 78, 119, 125, 127, 132, 135, 140]
Vitamin D^c^	6	171	310	3 studies [55, 130, 140]	–	2 studies [76, 139]
Magnesium^d^	8	288	541	3 studies [76, 77, 133]	1 study [139]	4 studies [78, 125, 132, 135]
Zinc^c, d^	8	286	345	2 studies [132, 133]	–	5 studies [71, 72, 76, 125, 135]
Iron^c, d^	10	420	383	1 study [132]	1 study [119]	7 studies [71, 127, 128, 133, 135, 141, 142]
Iodine	0	–	–	–	–	–

Notes: ^a^All DHA significance levels are for total plasma lipids or plasma phospholipids except for [53, 54] which reported erythrocyte lipid levels

^b^For [143], the statistical significance of a stated lower DHA level was not reported, therefore this study was not included in the outcomes above

^c^Differing findings for sub-grouped PKU only patients compared to controls reported for EPA [118], zinc [144], iron [145] and vitamin D [77]. These data are not included in this table. The data for all PKU patients from these publications were pooled for the meta-analysis

^d^For all studies by Schulpis et al. [124–128, 136], the PKU group was sub-grouped into well controlled PKU and poorly controlled PKU. Differences between well-controlled PKU and healthy controls only are reported in this table. The data for both groups of PKU patients were pooled for the meta-analysis

### Nutrients for which meta-analysis undertaken: DHA, EPA, cholesterol, zinc, vitamins B_12_, E and D, calcium, iron, magnesium, folate and selenium

**Fig. 2:**
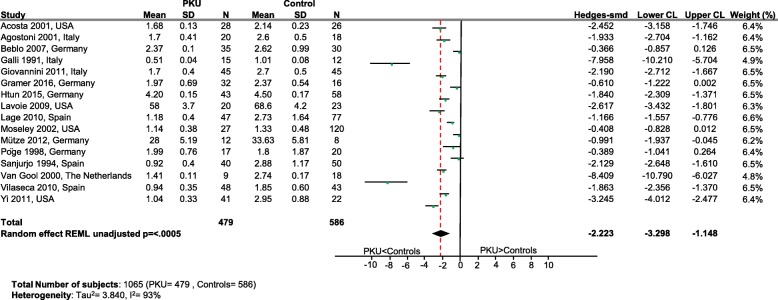
DHA levels in PKU patients versus healthy controls. Results of meta-analyses for DHA levels in PKU patients versus healthy controls. Abbreviations: DHA, docosahexaenoic acid; LCL, lower confidence limit; REML, restricted maximum likelihood; SD, standard deviation; SMD, standardized mean difference; UCL, upper confidence limit

**Fig. 3:**
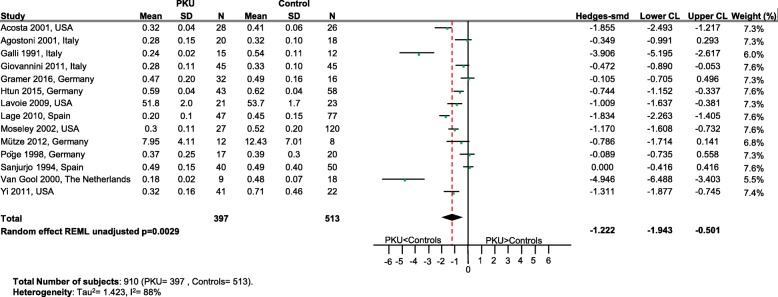
EPA levels in PKU patients versus healthy controls. Results of meta-analyses for EPA levels in PKU patients versus healthy controls. Abbreviations: EPA, eicosapentaenoic acid; LCL, lower confidence limit; REML, restricted maximum likelihood; SD, standard deviation; SMD, standardized mean difference; UCL, upper confidence limit

**Fig. 4:**
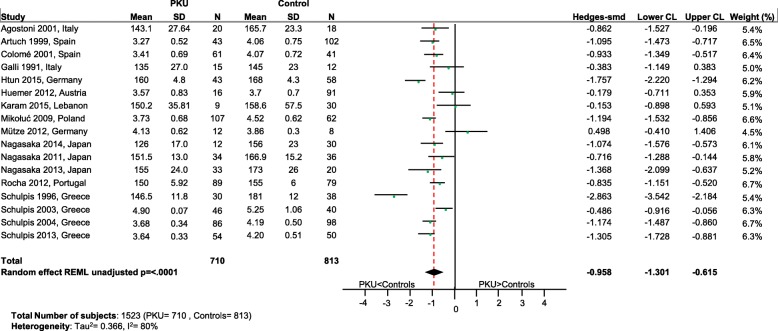
Cholesterol levels in PKU patients versus healthy controls. Results of meta-analyses for Cholesterol levels in PKU patients versus healthy controls. Abbreviations: LCL, lower confidence limit; REML, restricted maximum likelihood; SD, standard deviation; SMD, standardized mean difference; UCL, upper confidence limit

**Fig. 5:**
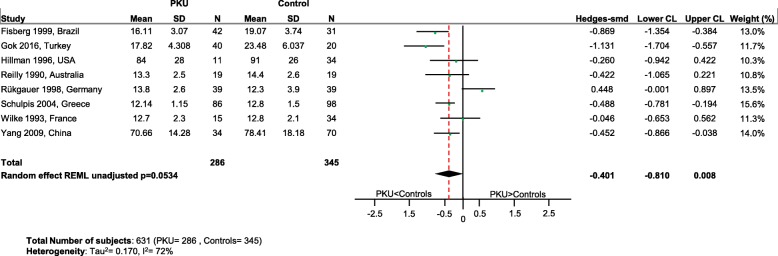
Zinc levels in PKU patients versus healthy controls. Results of meta-analyses for Zinc levels in PKU patients versus healthy controls Abbreviations: LCL, lower confidence limit; REML, restricted maximum likelihood; SD, standard deviation; SMD, standardized mean difference; UCL, upper confidence limit

Significantly lower levels of DHA (*p* = 0.0005; Fig. 2), EPA (*p* = 0.003; Fig. 3) and cholesterol (*p* < 0.0001; Fig. 4) were suggested in patients with PKU compared to healthy controls. Zinc levels tended to be lower in PKU patients compared to healthy controls, however this did not reach statistical significance but might be clinically relevant (*p* = 0.0534; Fig. 5).

**Fig. 6:**
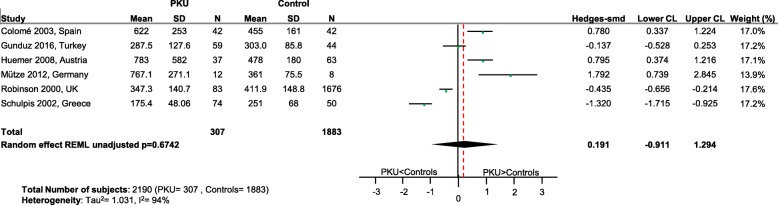
Vitamin B12 levels in PKU patients versus healthy controls. Results of meta-analyses for Vitamin B12 levels in PKU patients versus healthy controls. Abbreviations: LCL, lower confidence limit; REML, restricted maximum likelihood; SD, standard deviation; SMD, standardized mean difference; UCL, upper confidence limit

**Fig. 7:**
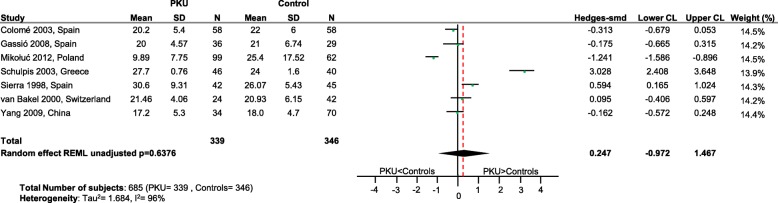
Vitamin E levels in PKU patients versus healthy controls. Results of meta-analyses for Vitamin E levels in PKU patients versus healthy controls. Abbreviations: LCL, lower confidence limit; REML, restricted maximum likelihood; SD, standard deviation; SMD, standardized mean difference; UCL, upper confidence limit

**Fig. 8:**
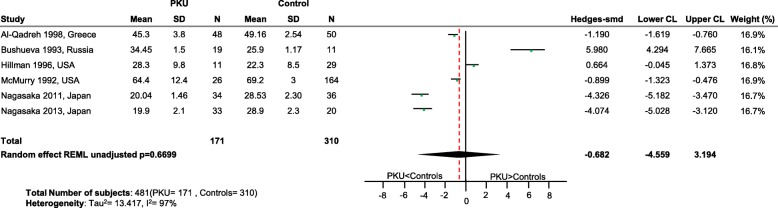
Vitamin D levels in PKU patients versus healthy controls. Results of meta-analyses for Vitamin D levels in PKU patients versus healthy controls. Abbreviations: LCL, lower confidence limit; REML, restricted maximum likelihood; SD, standard deviation; SMD, standardized mean difference; UCL, upper confidence limit

**Fig. 9:**
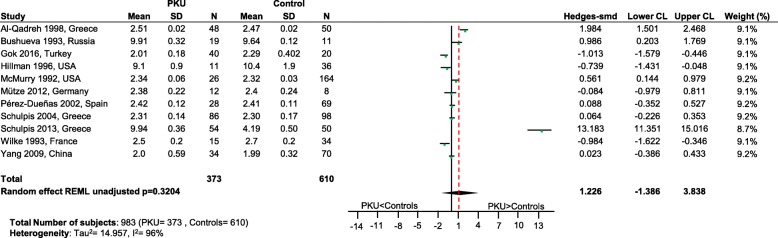
Calcium levels in PKU patients versus healthy controls. Results of meta-analyses for Calcium levels in PKU patients versus healthy controls Abbreviations: LCL, lower confidence limit; REML, restricted maximum likelihood; SD, standard deviation; SMD, standardized mean difference; UCL, upper confidence limit

**Fig. 10:**
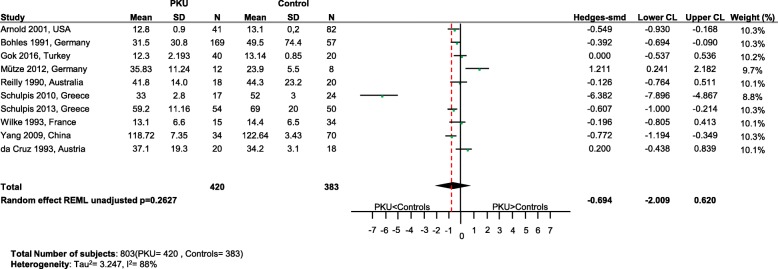
Iron levels in PKU patients versus healthy controls. Results of meta-analyses for Iron levels in PKU patients versus healthy controls. Abbreviations: LCL, lower confidence limit; REML, restricted maximum likelihood; SD, standard deviation; SMD, standardized mean difference; UCL, upper confidence limit

**Fig. 11:**
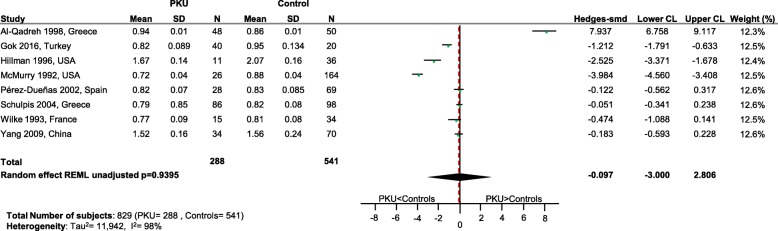
Magnesium levels in PKU patients versus healthy controls. Results of meta-analyses for Magnesium levels in PKU patients versus healthy controls. Abbreviations: LCL, lower confidence limit; REML, restricted maximum likelihood; SD, standard deviation; SMD, standardized mean difference; UCL, upper confidence limit

No differences in status between PKU patients and healthy controls were apparent for vitamins B_12_ (*p* = 0.67; Fig. 6), E (*p* = 0.64; Fig. 7) and D (p = 0.67; Fig. 8), calcium (*p* = 0.32; Fig. 9), iron (*p* = 0.26; Fig. 10) and magnesium (*p* = 0.94; Fig. 11).

**Fig. 12:**
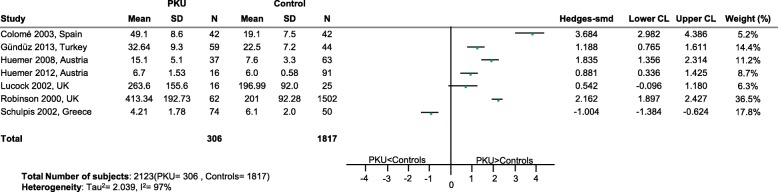
Folic Acid levels in PKU patients versus healthy controls. Folic Acid levels in PKU patients versus healthy controls. Abbreviations: LCL, lower confidence limit; REML, restricted maximum likelihood; SD, standard deviation; SMD, standardized mean difference; UCL, upper confidence limit

**Fig. 13:**
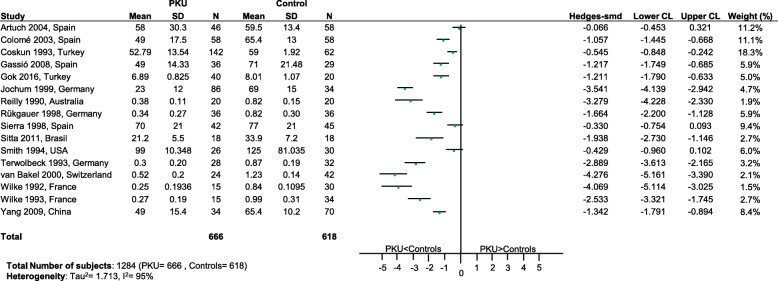
Selenium levels in PKU patients versus healthy controls. Selenium levels in PKU patients versus healthy controls. Abbreviations: LCL, lower confidence limit; REML, restricted maximum likelihood; SD, standard deviation; SMD, standardized mean difference; UCL, upper confidence limit

For folate (Fig. 12) and selenium (Fig. 13), the heterogeneity within the data was excessively high, so the overall conclusion of the meta-analysis was not reported. For selenium, whether the PS was supplemented or not varied for the seventeen studies identified. Therefore for studies (*n* = 5) where it was clearly stated in the publication that the PS was selenium-fortified [6, 30, 37, 44, 45], as there is less between-study variation, a further subgroup meta-analysis was undertaken for these five studies. This suggested that there was no difference in status between PKU patients taking a selenium-fortified PS compared to healthy controls (*p* = 0.0513; SMD: -0.61; 95% CI [− 1.23, 0.006]; 5 studies; 208 PKU patients; Tau^2^ = 0.193. It should be noted that four of the five studies in the subgroup analysis recruited subjects from the same European centre.

### Nutrients for which systematic review only undertaken (no meta-analysis possible): Vitamins A, C and B_6_, PLs, choline, iodine and uridine (forest plots presented as additional files)

As summarized in Table 3, for vitamins A (3 studies; Additional file 2) C (3 studies; Additional file 3), B_6_ (2 studies; Additional file 4) and PLs (2 studies; Additional file 5), an insufficient number of publications were retrieved to perform meta-analyses. No studies reporting plasma concentrations of choline, iodine or uridine status (plasma uridine or uridine monophosphate) in PKU were retrieved. However, two studies reported brain choline concentrations, which correlate with plasma choline levels, were found in PKU patients using proton magnetic resonance spectroscopy (1H-MRS), [46, 47]) (Additional file 6).

## Discussion

To the best of our knowledge, this is the first systematic review and meta-analysis of published studies investigating differences between PKU patients and healthy controls in the status of multiple nutrients involved in brain function. The nutrients investigated affect multiple structural and metabolic pathways within the brain and the potential implications of the altered nutrient status found or indeed the lack of data for some of these nutrients is discussed.

DHA, EPA, cholesterol, PL and choline are key structural components of neuronal membranes. Lower status of DHA, EPA and cholesterol levels are suggested by the meta-analyses for PKU patients compared to healthy controls. In healthy individuals, there is evidence of limited endogenous synthesis of DHA and EPA from the parent n-3 essential fatty acid, α linolenic acid [48–50] and in PKU patients the by-products of excessive phe may further inhibit synthesis of DHA [51]. Our findings of poorer DHA and EPA status concur with a previous meta-analysis carried out by Lohner et al. [36], who found suboptimal n-3 LC-PUFA status in PKU patients. As there have been many studies that have reported poor fatty acids status of PKU patients over the last 20 yrs, dietary advice has been modified and some PS have since been fortified with DHA, EPA and the parent essential fatty acids. Interestingly, some recent studies [52–54], where a few or all the patients were taking a fortified PS (essential or long chain n-3 fatty acids) or a modified fat diet, found no difference in DHA and EPA status in PKU patients compared to healthy controls.

Cholesterol is also a key component of neuronal membranes with 24S-hydroxycholesterol (24 s-OHC) [25] a specific metabolite of brain cholesterol metabolism measurable in blood. Decreased levels of plasma 24 s-OHC in PKU individuals have been reported which may indicate reduced cholesterol synthesis in the brain and/or disturbed conversion from cholesterol to 24S-OHC [55]. Some animal studies have reported that cholesterol synthesis maybe suppressed in PKU with reduced activity of two key cholesterol synthesis enzymes [55]. Furthermore, Colome et al., 2003 [44] reported a relationship between high plasma phenylalanine levels and an inhibition of cholesterogenesis, although the low fat and saturates intakes typical in PKU diets may also result in lower serum cholesterol levels. The finding of significantly lower total plasma cholesterol levels in PKU patients in this meta-analysis may suggest altered brain levels and given the importance of this nutrient for brain function, further studies directly measuring 24 s-OHC in patients with PKU should be considered.

A phe-dependent down regulation of the hydroxymethylglutaryl (HMG)-CoA reductase and the mevalonate-5-pyrophosphate decarboxylase activities, may potentially reduce the synthesis of both cholesterol and coenzyme Q10 (CoQ10) through their common mevalonate pathway [56–58]. CoQ10, a lipid component acting as an electron carrier in the mitochondrial respiratory chain, has been negatively correlated with phe levels in serum of PKU patients [59–61]. However, it is suggested that that HPA has less profound effect upon CoQ10 synthesis than cholesterol synthesis [57, 62] in PKU low plasma levels of CoQ10 may be caused by increased oxidative stress [45].

Only two studies reporting on PL status were identified; Galli et al. reported no significant difference in 15 children with PKU compared to controls and Pietz et al., using ^31^P-MR spectroscopy [34] reported significantly lower baseline brain membrane bound PL levels, but no differences in mobile PL or catabolic metabolites in 11 PKU patients.

Antioxidant nutrients such as zinc, selenium and vitamins A, C and E play a key role in detoxifying reactive intermediates in the brain. Where intakes of antioxidant nutrients are poor, such as in PKU, antioxidant defenses may be compromised, contributing to the highly oxidative environment observed in PKU. Increased markers of oxidative stress have been observed in PKU populations; for example, Colome et al. [44] found plasma lipid peroxidation to be increased in PKU and Artuch et al. [45] observed that ubiquinone-10 concentrations were significantly lower in PKU patients compared with healthy controls. High levels of circulating phe are known to exacerbate excessive production of reactive oxygen species [63]. It is suggested that systemic oxidative stress causes increased lipid peroxidation and altered plasmatic fatty acid profiles [64]. Also associated with a high number of double bonds, DHA/EPA are particularly susceptible to peroxidative breakdown, offering an additional explanation for the lower levels of these fatty acids as found in the current meta-analysis.

For the antioxidant nutrients, we found no apparent difference in status for vitamin E and zinc, too few studies identified to carry out meta-analyses for vitamins A and C, or the degree of heterogeneity was too high to draw a definitive conclusion for selenium. Selenium is a nutrient of particular concern in PKU given that dietary sources are very limited, and intakes are typically very poor when compliance with the PS prescription is inadequate. The selenium content of vegetation varies depending on the concentrations in soil, with Europe and China typically having lower concentrations than North America [65]. Selenium supplementation of PKU diets was introduced in the mid-1980s [6] when selenium essentiality became more widely accepted. Although there may be a suggestion from the limited cluster analysis in this review and evidence from intervention studies [66, 67] that selenium supplementation is effective in improving status, it is also observed that 14 of the 16 studies in this review had confidence intervals on the left side of the plot, suggesting lower levels in PKU patients compared to controls. Therefore, although improvement in selenium status is observed in clinical practice with supplementation, the findings from this meta-analysis are inconclusive because of the high heterogeneity within the data. This may be due to several factors including: variable selenium intakes depending on whether the PS was fortified/not over the timeframe of the literature review; variation in patient compliance with PS over a wide age range and bioavailability of different sources of selenium [68] and/or low concentrations of selenium in grains and vegetables (many studies identified were in European populations). Furthermore, both the PKU and the healthy control groups in the studies were found to have selenium status that was low or close to lower European population norms in some studies [44, 45, 67, 69–74] Therefore, at this time further research is warranted investigating selenium status in PKU and the effects of supplementation including measurement of plasma selenoprotein P (SEPP1) as a more informative marker of status.

Adherence to the phe-restricted diet is necessary to achieve plasma phe control including compliance with PS as the predominant source of many nutrients in the PKU diet, such as vitamin B_12_, calcium, vitamin D, zinc and iron and to ensure adequate micronutrient status. However, patients who are non- or poorly- compliant with their PS prescription are at greatest risk of suboptimal intakes of these nutrients. For example, there have been several case studies reporting poor vitamin B_12_ status in patients as a consequence of a relaxed PKU diet [7, 75] and low intakes of calcium have been reported in patients with PKU [76–78] particularly if compliance is poor in older children/adults. The meta-analysis found no difference in plasma calcium levels in PKUs compared to healthy controls; however, plasma calcium is not a suitable biomarker of status [79]. So no conclusion about the status of calcium related to brain function in PKU can be drawn from this finding. We did not find a between group difference in vitamin D status between PKUs and healthy controls, however, minimal cutaneous synthesis is now assumed and recommended dietary intakes for vitamin D have increased globally in recent years [80, 81]. There is also emerging knowledge of the role of vitamin D as a ‘neuro-steroid’ and regulator of brain serotonin synthesis [82, 83]. Given this increasing knowledge on vitamin D requirements, dietary intakes of vitamin D by those with PKU should be monitored to ensure recommended intakes are achieved.

Intakes of folic acid are much less likely to be compromised given that good food sources, such as fruit and vegetables, are allowed in controlled or unrestricted quantities in the diet and that PS are routinely supplemented. In the meta-analysis for folic acid the excessively high heterogeneity across the studies undermined its reliability to draw a conclusion on between group differences in status. Nonetheless 6 of the 7 studies [84–89] had confidence intervals on the right side of the plot (Fig. 6), suggesting positive differences between PKU patients and healthy controls. Intakes of folic acid by patients who are compliant with their PS prescription may be higher than desired when combined with intakes from normal foods that are also good sources of folic acid, and indeed high serum folate levels have been observed in PKU [90–92]. Vitamin B_12_ deficiency may be masked where circulating folate levels are high [93] and Walter et al. [9] reported that functional vitamin B_12_ deficiency can occur in the presence of normal B_12_ concentrations in PKU. Although we observed no apparent difference in vitamin B_12_ status in PKU patients compared to healthy controls suggesting that supplementation is effective in maintaining nutrient status comparable to controls, the physiological effect of potentially high folate intakes and levels needs consideration.

The systematic review found limited or no publications matching our entry criteria on nutritional status of some nutrients (vitamins A, C, B_6_, choline, uridine, iodine and PL). Although the status of some nutrients such as vitamin C is unlikely to be compromised in PKU, others such as choline and iodine warrant consideration. In healthy individuals, de-novo synthesis of choline is not sufficient to meet requirements and recommended or adequate intakes (AI) have been established [94, 95], however, sub-optimal intakes of choline are reported in the general population compared to the recommended AI [96, 97]. The main choline-rich food groups are restricted in PKU, and many PS are supplemented with choline. Dezortova et al. reported significantly lower brain choline concentrations (*p* < 0.05) in 15 adult PKU patients on a moderate/strict diet compared to controls [33]. Whereas, Sijens [98] reported no significant difference in brain choline levels between 10 adult PKU patients (including both compliant and non-compliant) and those of healthy controls. No detail on dietary intake of choline was provided in these studies. Because of the wide-ranging role of choline in brain function, if present, inadequate choline status in PKU may have an impact on neurological outcomes and so dietary intakes should be monitored and studies investigating choline status should be considered. This systematic review revealed no publications matching our inclusion criteria comparing iodine status of PKU patients with healthy controls. However, in PKU, an altered free thyroid hormone concentrations in PKU, in association with an impaired activity of thyroxine-5-deiodinase enzyme is described [35, 99],

Given that intakes of iodine from normal foods in PKU diets are likely to be very low and that iodine has an important role in thyroid function and brain development [100], the possibility of insufficiency among poorly compliant patients in particular should be considered.

### Limitations and recommendations

The robustness of the conclusions drawn from any systematic review and meta-analysis is limited by the quality, quantity and content of the available data. In addition, the relationship between nutrient intakes and status is complex, and depends on many factors such as the bioavailability of nutrients, nutrient interactions and individual metabolism. Although these meta-analyses provide valuable insight into the status of nutrients, there was a high degree of within- and between- study heterogeneity. This may be due to a number of factors such as: variation in nutrient intake; historical differences in the fortification of PS; degree of dietary compliance; the wide age range of patients affecting compliance; broad phe tolerance determining natural food intakes and changes in management during the 25 years of literature included in this review. Also, there was a lack of information in several papers on dietary intake and compliance including nutrient fortification of the PS/not. Another limitation is that over the timeframe of the systematic review, improved markers of nutritional status have been identified for example, all of the studies reported plasma selenium concentrations as the status marker, whereas more recently plasma selenoprotein P (SEPP1) has been considered to be a more informative marker of status. Comparison with healthy controls assumes that their nutritional status is adequate however for some nutrients e.g. iron or vitamin D, status can be compromised in otherwise healthy populations [101].

As for all studies measuring nutritional status, it is important that the most relevant biomarkers are measured, and sampling procedures strictly followed. It is recommended that more details of patient characteristics (severity of PKU) and dietary information (details of protein tolerance, PS dosage, BH4 intake and adherence) is reported in studies. Given the small numbers of patients available for study in PKU and in the absence of multi-centre studies, systematically reviewing the literature and undertaking meta-analysis to inform and support best practice is a valuable tool. To allow extraction of data on key items from studies for future meta-analyses, we recommend that a standardised approach for the reporting of observational studies should be followed as this would allow more robust and definitive conclusions to be drawn from meta-analyses.

### Concluding remarks

Whilst changes in fortification practices of PS and dietary advice more generally have led to improvements in status for some of the nutrients reviewed, to ensure optimal outcome in PKU, patients’ micronutrient and fatty acid status should continue to be monitored, with a particular focus on patients who are non-compliant or poorly compliant with their PS. We noted that for several of the nutrients we identified as being of interest in this review, only a few or no publications matching our inclusion criteria were found. We expect that for those nutrients for which patients depend almost exclusively on their PS, such as choline and iodine, nutrient insufficiency among poorly compliant PKU patients should be explored. As is standard practice, the importance of dietary compliance should continue to be emphasized to patients including adherence to the PS as an invaluable source of micronutrients. Dietary management is complex and changing with the advent of new pharmaceutical adjunctive therapies (e.g tetrahydrobiopterin and phenylalanine ammonium lysase) where a more liberal diet with more natural protein may be taken, for these patient groups it is important to ensure that there is an ongoing adequate intake of a range of micronutrients. Although the meta-analyses revealed differences between healthy controls and PKU patients in nutrient status for some of nutrients, we did not investigate whether poor status has a role in the pathophysiology of any of the neurological deficits observed in treated PKU patients. However, status of key nutrients known to have a role in cognitive development and functioning should be monitored.

Our recommendations for the biochemical monitoring for relevant nutrients for PKU brain function (using the European PKU guidelines [2] and the PKU Nutrition Management Guidelines of the Southeast Regional Newborn Screening Collaborative (SERC) and Genetic Metabolic Dietitians International (GMDI) [102] for direction) are given in Table 4.

**Table 4:** Assessment of Biochemical Status for nutrients/metabolites

Routinely	Annual^a, b^, biannual^b^	Haemoglobin, mean corpuscular volume, and ferritin^a, b^; micronutrients (vitamins and minerals including: calcium, zinc, and selenium (SEPP1)) or hormones (parathyroid hormone) if clinically indicated^a^ Vitamin D 25-OH^b^ Our recommendation: DHA^c^, EPA^c^, Cholesterol
Conditional^c^,^b^	Annual or as indicated^b^	Vitamin B_12_ (plasma methylmalonic acid, total homocysteine), erythrocyte folate, zinc, copper, essential fatty acids (linoleic acid and alpha-linolenic acid).
	Every 5 years beginning with baseline at age 5^b^	Bone density (DXA scan)^b^ Bone mineral density (BMD)^a^ during late adolescence; if abnormal, the measurement should be repeated after 1 year

^a^ [2], ^b^ [102] excluding maternal PKU

^3^Monitoring is indicated by poor adherence to prescribed therapy (diet, medical food, or pharmacotherapy), clinical symptoms of nutritional inadequacy are present (e.g. poor growth), or serious intercurrent illness

## Additional files

Additional file 1: Search Strategy (PDF 244 kb).

Additional file 2: Vitamin A levels in PKU patients versus healthy controls. Vitamin A levels in PKU patients versus healthy controls. Abbreviations: LCL, lower confidence limit; REML, restricted maximum likelihood; SD, standard deviation; SMD, standardized mean difference; UCL, upper confidence limit (PDF 271 kb).

Additional file 3: Vitamin C levels in PKU patients versus healthy controls. Vitamin C levels in PKU patients versus healthy controls. Abbreviations: LCL, lower confidence limit; REML, restricted maximum likelihood; SD, standard deviation; SMD, standardized mean difference; UCL, upper confidence limit (PDF 169 kb).

Additional file 4: Vitamin B6 levels in PKU patients versus healthy controls. Vitamin B6 levels in PKU patients versus healthy controls. Abbreviations: LCL, lower confidence limit; REML, restricted maximum likelihood; SD, standard deviation; SMD, standardized mean difference; UCL, upper confidence limit (PDF 165 kb).

Additional file 5: Phospholipid levels in PKU patients versus healthy controls. Phospholipid levels in PKU patients versus healthy controls. Abbreviations: LCL, lower confidence limit; REML, restricted maximum likelihood; SD, standard deviation; SMD, standardized mean difference; UCL, upper confidence limit (PDF 166 kb).

Additional file 6: Choline levels in PKU patients versus healthy controls. Choline levels in PKU patients versus healthy controls. Abbreviations: LCL, lower confidence limit; REML, restricted maximum likelihood; SD, standard deviation; SMD, standardized mean difference; UCL, upper confidence limit (PDF 166 kb).

## Abbreviations

BMD: Bone mineral density
CoQ10: Coenzyme Q10
DHA: Docosahexaenoic acid
EPA: Eicosapentanoic acid
HMG-CoA: Hydroxymethylglutaryl coenzyme A
HPA: Hyperphenylalaninaemia
LCPUFA: Long chain polyunsaturated fatty acids
phe: Phenylalanine
PKU: Phenylketonuria
PLs: Phospholipids
PRISMA: Preferred Reporting Items for Systematic Reviews and Meta-Analyses
PS: Protein substitute
REML: Restricted maximum likelihood estimation
SEPP1: Selenoprotein P
SMD: Standardized mean difference
trp: Tryptophan
tyr: Tyrosine
