# Supplementary material for: Status of nutrients important in brain function in phenylketonuria: a systematic review and meta-analysis
Source: Orphanet J Rare Dis. 2018 Jun 26;13:101. doi: 10.1186/s13023-018-0839-x (PMC6020171; doi:10.1186/s13023-018-0839-x)
Supplement: Supplementary file 1 — Search Strategy (PDF 244 kb). [file 13023_2018_839_MOESM1_ESM.pdf]

Additional file 1. Search strategy

The search strategy searched for the following terms in the title, abstract, or descriptors: TI,AB,SU(((phenyl p/0 ketonuri*) OR phenylketonuri* OR PKU OR hyperphenylalanin* OR (phenylalanine p/0 deficien*) OR ((pah or (phenylalanine p/0 hydroxylase)) p/0 deficien*)) AND (EPA OR LCPUFA OR LCPUFAs OR PUFA OR PUFAs OR (fatty p/0 acids) OR (fatty p/0 acid) OR (Eicosapentaenoic p/0 acid) OR (Omega-3-eicosapentaenoic p/0 acid) OR (Timnodonic p/0 acid) OR DHA OR (docosahexaenoic p/0 acid) OR (cervonic p/0 acid) OR phospholipid* OR phosphatide* OR choline OR uridine OR (uridylic p/0 acid) OR UMP OR (uridine p/0 monophosphate) OR (vitamin p/0 E) OR (alpha-tocopherol p/0 equivalents) OR tocopherol OR (antioxidant p/0 status) OR (oxidative p/0 stress) OR (vitamin p/0 C) OR (ascorbic p/0 acid) OR ascorbate OR (vitamin p/0 B12) OR cobalamin OR (vitamin p/0 B6) OR pyridoxine OR (pyridoxal p/0 phosphate) OR (folic p/0 acid) OR (vitamin p/0 B9) OR (vitamin p/0 B11) OR (vitamin p/0 M) OR selenium OR Folacin OR Folate OR (Pteroyl-L-glutamic p/0 acid) OR (Pteroyl-L-glutamate) OR calcium OR "Ca" OR "Vitamin D" OR "vitamin D3" OR colecalciferol OR ergocalciferol OR "1,25-dihydroxycholecalciferol" OR "1,25(OH)2D" OR Magnesium OR "Mg" OR Zinc OR "Zn" OR Iron OR "Fe" OR Iodine OR Cholesterol OR "(3β)-cholest-5-en-3-ol" OR Cholesterin OR "Cholesteryl alcohol" OR Copper OR "Cu" OR Manganese OR "Mn" OR "Vitamin A" OR Retinol OR Carotene OR Carotenoid OR (Retinyl p/0 ester*) OR Retinal OR (Retinoic p/0 Acid) OR (retinyl p/0 palmitate)))
