# Supplementary figures and images for: Status of nutrients important in brain function in phenylketonuria: a systematic review and meta-analysis
Source: Orphanet J Rare Dis. 2018 Jun 26;13:101. doi: 10.1186/s13023-018-0839-x (PMC6020171; doi:10.1186/s13023-018-0839-x)

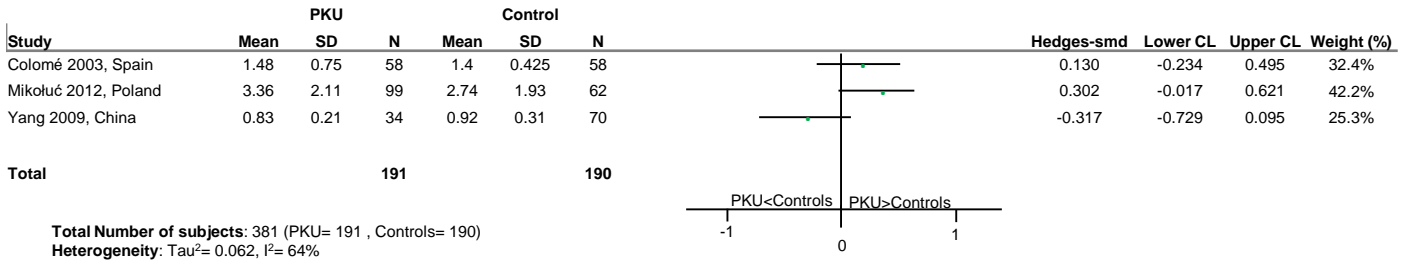

Supplement: Supplementary file 2 — Vitamin A levels in PKU patients versus healthy controls. Vitamin A levels in PKU patients versus healthy controls. Abbreviations: LCL, lower confidence limit; REML, restricted maximum likelihood; SD, standard deviation; SMD, standardized mean difference; UCL, upper confidence limit (PDF 271 kb). [file 13023_2018_839_MOESM2_ESM.pdf]

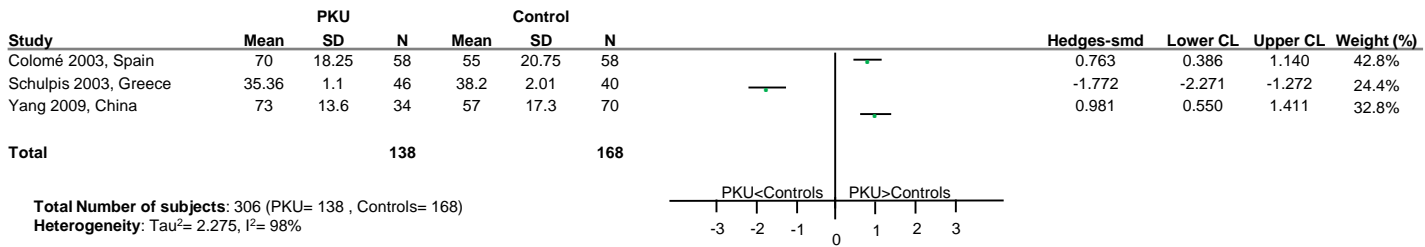

Supplement: Supplementary file 3 — Vitamin C levels in PKU patients versus healthy controls. Vitamin C levels in PKU patients versus healthy controls. Abbreviations: LCL, lower confidence limit; REML, restricted maximum likelihood; SD, standard deviation; SMD, standardized mean difference; UCL, upper confidence limit (PDF 169 kb). [file 13023_2018_839_MOESM3_ESM.pdf]

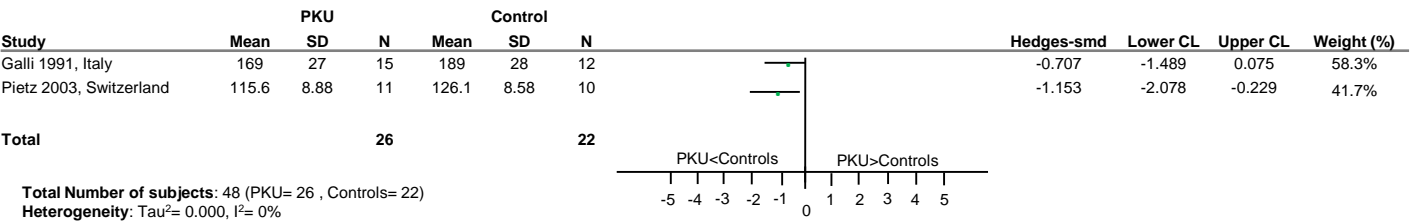

Supplement: Supplementary file 5 — Phospholipid levels in PKU patients versus healthy controls. Phospholipid levels in PKU patients versus healthy controls. Abbreviations: LCL, lower confidence limit; REML, restricted maximum likelihood; SD, standard deviation; SMD, standardized mean difference; UCL, upper confidence limit (PDF 166 kb). [file 13023_2018_839_MOESM5_ESM.pdf]

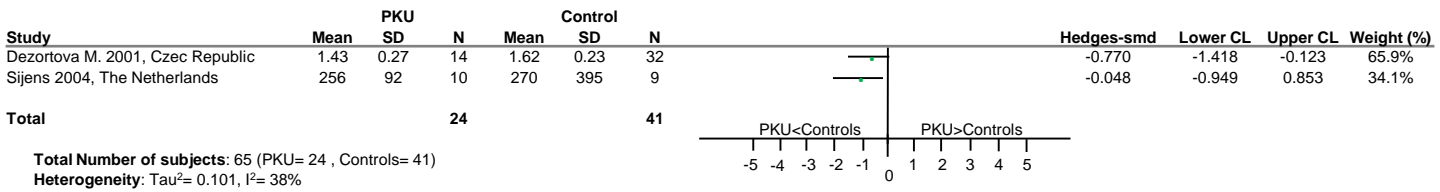

Supplement: Supplementary file 6 — Choline levels in PKU patients versus healthy controls. Choline levels in PKU patients versus healthy controls. Abbreviations: LCL, lower confidence limit; REML, restricted maximum likelihood; SD, standard deviation; SMD, standardized mean difference; UCL, upper confidence limit (PDF 166 kb). [file 13023_2018_839_MOESM6_ESM.pdf]
