# Supplementary material for: Status of nutrients important in brain function in phenylketonuria: a systematic review and meta-analysis
Source: Orphanet J Rare Dis. 2018 Jun 26;13:101. doi: 10.1186/s13023-018-0839-x (PMC6020171; doi:10.1186/s13023-018-0839-x)
Supplement: Supplementary file 4 — Vitamin B6 levels in PKU patients versus healthy controls. Vitamin B6 levels in PKU patients versus healthy controls. Abbreviations: LCL, lower confidence limit; REML, restricted maximum likelihood; SD, standard deviation; SMD, standardized mean difference; UCL, upper confidence limit (PDF 165 kb). [file 13023_2018_839_MOESM4_ESM.pdf]

	PKU			Control						
**Study**	**Mean**	**SD**	**N**	**Mean**	**SD**	**N**	**Hedges-smd**	**Lower CL**	**Upper CL**	**Weight (%)**
Prince 1994, USA	99.4	54.0	15	53.5	21.3	6	0.925	-0.062	1.913	20.2%
Schulpis 2002, Greece	36.7	7.24	74	60.2	10	50	-2.761	-3.258	-2.265	79.8%
**Total**			**89**			**56**				

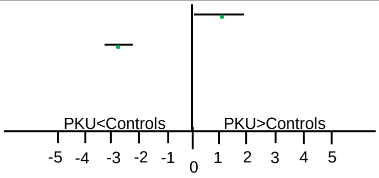

Total Number of subjects: 145 (PKU= 89 , Controls= 56)
Heterogeneity: Tau^2= 6.637, I^2= 98%
